# Rho1 GTPase and PKC Ortholog Pck1 Are Upstream Activators of the Cell Integrity MAPK Pathway in Fission Yeast

**DOI:** 10.1371/journal.pone.0088020

**Published:** 2014-01-31

**Authors:** Laura Sánchez-Mir, Teresa Soto, Alejandro Franco, Marisa Madrid, Raúl A. Viana, Jero Vicente, Mariano Gacto, Pilar Pérez, José Cansado

**Affiliations:** 1 Yeast Physiology Group, Department of Genetics and Microbiology, Facultad de Biología, Universidad de Murcia, Murcia, Spain; 2 Instituto de Biología Funcional y Genómica, Consejo Superior de Investigaciones Científicas/Departamento de Microbiología y Genética, Universidad de Salamanca, Salamanca, Spain; Institute of Biology Valrose, France

## Abstract

In the fission yeast *Schizosaccharomyces pombe* the cell integrity pathway (CIP) orchestrates multiple biological processes like cell wall maintenance and ionic homeostasis by fine tuning activation of MAPK Pmk1 in response to various environmental conditions. The small GTPase Rho2 positively regulates the CIP through protein kinase C ortholog Pck2. However, Pmk1 retains some function in mutants lacking either Rho2 or Pck2, suggesting the existence of additional upstream regulatory elements to modulate its activity depending on the nature of the environmental stimulus. The essential GTPase Rho1 is a candidate to control the activity of the CIP by acting upstream of Pck2, whereas Pck1, a second PKC ortholog, appears to negatively regulate Pmk1 activity. However, the exact regulatory nature of these two proteins within the CIP has remained elusive. By exhaustive characterization of strains expressing a hypomorphic Rho1 allele (*rho1-596*) in different genetic backgrounds we show that both Rho1 and Pck1 are positive upstream regulatory members of the CIP in addition to Rho2 and Pck2. In this new model Rho1 and Rho2 control Pmk1 basal activity during vegetative growth mainly through Pck2. Notably, whereas Rho2-Pck2 elicit Pmk1 activation in response to most environmental stimuli, Rho1 drives Pmk1 activation through either Pck2 or Pck1 exclusively in response to cell wall damage. Our study reveals the intricate and complex functional architecture of the upstream elements participating in this signaling pathway as compared to similar routes from other simple eukaryotic organisms.

## Introduction

Studies on molecular clues involved in the regulation MAPK signaling pathways are essential to understand how eukaryotic cells are able to adapt and survive against suboptimal environmental conditions. The rod-shaped, fission yeast *Schizosaccharomyces pombe* is an excellent model organism to study mechanisms and cellular events linked to MAPK activation, given the significant functional homology between their regulatory circuits and those of higher cells [1], [2]. The cell integrity pathway (CIP), one of the three MAPK pathways present in fission yeast, regulates multiple processes like cell wall construction and maintenance during stress, vacuole fusion, cytokinesis, morphogenesis, and ionic homeostasis through its central element, MAPK Pmk1 [3]–[8]. Pmk1 is ortholog to human ERK1/2 and associates *in vivo* with MAPKKK Mkh1and MAPKK Pek1 to form a ternary complex [9]–[12], becoming activated in response to multiple adverse conditions such as hyper- and hypo-osmotic stress, glucose withdrawal, cell wall damage, and oxidative stress induced by hydroperoxides or pro-oxidants [12]. Importantly, *S. pombe* mutants lacking Mkh1, Pek1, or Pmk1 display strong sensitivity to the above stresses [12], indicating that a functional MAPK module is required for cell adaptation and survival under such conditions.

Previous work demonstrated that Rho2 GTPase, one of the six Rho GTPases found in *S. pombe* proteome (Rho1 to Rho5, and Cdc42) which controls cell polarity and cell wall biosynthesis, is a positive regulator operating upstream of the CIP [13], [14]. Rho2-dependent regulation of Pmk1 activity is mediated through Pck2, one of the two orthologs of protein kinase C (PKC) present in this organism [13], [14]. On the contrary, Pck1, the second PKC ortholog, appears to negatively regulate the activity of the CIP by an unknown mechanism, since Pck1-less mutants display a moderate increase in basal Pmk1 phosphorylation [14]. Notably, simultaneous deletion of Pck1 and Pck2 is lethal, suggesting that both kinases share a functional role that is essential during fission yeast growth [15]. Importantly, we demonstrated that Pmk1 can still be activated in the absence of either Rho2 or Pck2, supporting the existence of a complex scenario where several routes involving various (known and unknown) elements regulate Pmk1 activation depending on the nature of the activating stimulus [14]. This model is in striking contrast to the situation in budding yeast *Saccharomyces cerevisiae*, where RHO1 GTPase and PKC1 (ortholog to both Pck1 and Pck2) are absolutely needed for the activation of MAPK SLT2/MPK1 [16].

While Cdc42 GTPase does not appear to be involved in Pmk1 activation [12], Rho1, another essential GTPase, has emerged as a strong candidate to participate as an alternative/additional upstream activator of the CIP, since some of the phenotypes associated to lack of Rho2 are partially rescued by Rho1 [17]. Moreover, both GTP-bound Rho1 and Rho2 interact *in vivo* with either Pck1 or Pck2 [18], [19], and target Pck2 to coordinately regulate the biosynthesis of (1,3) β-D-glucan (Rho1) and α-glucan (Rho2), the two main cell wall polymers in fission yeast [20]. Additionally, results obtained after the characterization of mutants lacking Rgf1, which is the main Guanine Nucleotide Exchange Factor (GEF) involved in Rho1 activation *in vivo*, has led to the hypothesis that this GTPase regulates Pmk1 activation via Pck2 [21]. The evidences obtained included the observation that Pmk1 activation in response to stress is fully (hyper- and hypo-osmotic treatment) or partially (cell wall damage) abrogated in Rgf1-less cells, and that overexpression of a hyperactive version of Rho1 induced a clear increase in MAPK phosphorylation which was suppressed in the absence of Pck2 [21]. Nevertheless, the promiscuous nature of GEFs together with the fact that Rho1 deletion is lethal has complicated the elucidation of the specific roles of this GTPase as an upstream activator of the CIP. In this context, we have recently described the isolation of a fission yeast mutant expressing a genomic hypoactive version of Rho1 with reduced GTPase activity *in vivo* (*rho1-596* allele) [22]. *rho1-596* cells are viable but show severe cell wall defects and a thermosensitive phenotype when incubated above 34°C [22]. Importantly, Pmk1 basal activity is increased in this mutant, and deletion of either Rho2 or Pmk1 partially rescued *rho1-596* thermosensitivity [22], supporting the existence of a complex functional relationship between Rho1 and Rho2 during downstream signalling to the CIP. In this study we show for the first time that Rho1 and Pck1 are true activators of this signalling cascade in addition to Rho2 and Pck2 under specific environmental contexts.

## Materials and Methods

### Strains, plasmids and growth conditions

The *S. pombe* strains (Table 1) were grown with shaking at 28°C in either YES or EMM2 medium with 2% of glucose, and supplemented with adenine, leucine, histidine or uracil (100 mg/liter, Sigma Chemical) [23]. Mutant strains were obtained by standard transformation procedures or by mating and selecting diploids in EMM2 medium without supplements. Spores were obtained in MEL medium, purified by glusulase treatment and allowed to germinate in EMM2 plus the appropriate requirements [24]. Transformation of yeast strains was performed by the lithium acetate method [19]. Plasmids pREP41X-*rho1^+^*(*G15V*) and pREP3X-*rho2^+^* express, respectively, hyperactive and wild type alleles of Rho1 and Rho2 GTPases [15], [20] under the control of the attenuated (41X) and strong (3X) versions of the thiamine-repressible promoter *nmt1* [25]. Plasmid pREP41X-*pck1^+^* was used to express a wild type version of PKC-type kinase Pck1 [15]. To construct strains expressing C-terminal 13myc-tagged versions of *mkh1^+^* we employed plasmid pFA6a-13myc-kanMX6 [26].

**Table 1:** *S. pombe* strains.

Strain^a^	Genotype	Source/Reference
MI200	h^+^ *pmk1-HA6H::ura4^+^*	Madrid *et al* (2006)
MI102	h^+^ *pmk1::kanMX6*	Madrid *e tal* (2006)
YFG35	h^−^ *pck1::ura4^+^*	Lab stock
MI700	h^+^ *rho2:: kanMX6 pmk1-HA6H:: ura4^+^*	Barba *et al.* (2008)
GB3	h^+^ *pck2:: kanMX6 pmk1 HA6H:: ura4^+^*	Barba *et al.* (2008)
GB29	h^−^ *rho2:: kanMX6 pck2:: kanMX6 pmk1- HA6H:: ura4^+^*	Barba *et al.* (2008)
GB35	h^+^ *pck1::ura4^+^ pmk-HA6H::ura4^+^*	Barba *et al.* (2008)
LS201	h^−^ *rho1-596::NatMX6 pmk1-HA6H:: ura4^+^*	Viana *et al.* (2013)
LS209	h^+^ *rho1-596::NatMX6 pmk1::kanMX6*	This work
LS202	h^+^ *rho1-596::NatMX6 rho2:: kanMX6 pmk1-HA6H:: ura4^+^*	Viana *et al.* (2013)
LS203	h^+^ *rho1-596::NatMX6 pck2:: kanMX6 pmk1-HA6H:: ura4^+^*	Viana *et al.* (2013)
LS204	h^+^ *rho1-596::NatMX6 pck1:: ura4^+^ pmk1-HA6H:: ura4^+^*	Viana *et al.* (2013)
LS206	h^+^ *rho2:: kanMX6 pck1::ura4^+^ pmk1- HA6H:: ura4^+^*	This work
LS207	h^+^ *rho1-596::NatMX6 rho2:: kanMX6 pck2:: kanMX6 pmk1-HA6H:: ura4^+^*	This work
LS208	h^+^ *rho1-596::NatMX6 rho2:: kanMX6 pck1:: ura4^+^ pmk1-HA6H:: ura4^+^*	This work
PPG541	h^−^ *nmt41:HA-pck1::ura4^+^*	Lab stock
LS210	h^+^ *mkh1-13myc:: kanMX6*	This work
LS211	h^−^ *nmt41:HA-pck1::ura4^+^ mkh1-13myc:: kanMX6*	This work
LS212	h^−^ *nmt41:HA-pck1::ura4^+^ pck2:: kanMX6 mkh1-13myc:: kanMX6*	This work
LS213	h^−^ *nmt41:HA-pck1::ura4^+^ pck2:: kanMX6*	This work

a All strains are *ade- ura4D-18 leu1-32*.

### Stress treatments and detection of activated Pmk1

Experiments to investigate Pmk1 activation under stress were made using log-phase cell cultures (OD_600_ = 0.7) growing at 28°C in YES, and supplemented with either Caspofungin (Merck), Calcofluor (Sigma Chemical), potassium chloride (Sigma Chemical), or hydrogen peroxide (Sigma Chemical). In glucose deprivation studies, cells were grown in YES medium with 7% glucose to an OD_600_ = 0.5, recovered by filtration, and resuspended in the same medium without glucose but osmotically equilibrated with 3% glycerol [12]. At different times, cells from 50 ml of culture were harvested by centrifugation at 4°C, washed with cold PBS buffer, and the yeast pellets immediately frozen in liquid nitrogen for analysis. Cell homogenates were prepared under native conditions employing chilled acid-washed glass beads and lysis buffer (10% glycerol, 50 mM Tris-HCl pH 7.5, 150 mM NaCl, 0.1% Nonidet NP-40, plus specific protease and phosphatase inhibitor, Sigma Chemical). The lysates were cleared by centrifugation at 13000 rpm for 15 min, and the proteins were resolved in 10% SDS-PAGE gels, transferred to nitrocellulose filters, and incubated with rabbit anti-phospho-p42/44 antibodies (Cell Signalling) [12]. The immunoreactive bands were revealed with anti-rabbit HRP-conjugated secondary antibodies (Sigma Chemical) and the ECL detection kit (GE Healthcare). Densitometric quantification of Western blot signals was performed using ImageJ software [27].

### Immunoprecipitation

Cell extracts (4 mg total protein) were obtained using lysis buffer (50 mM Tris/HCl pH 7.5, 0.5% sodium deoxicholate, 150 mM NaCl, 1% NP-40 and protease inhibitor (Sigma Chemical), and incubated with anti-HA monoclonal antibody and protein A-sepharose beads for 4 h at 4°C. The beads were washed two times with lysis buffer, two times with washing buffer 2 (50 mM Tris/HCl pH 7.5, 0.05% sodium deoxicholate, 500 mM NaCl, 0.1% NP-40), one time with washing buffer 3 (50 mM Tris/HCl pH 7.5, 0.05% sodium deoxicholate, 0.1% NP-40), and resuspended in sample buffer. Proteins were separated in 8% SDS-PAGE gels, transferred to nitrocellulose filters (GE Healthcare), and hybridized with either anti-HA or anti-c-myc (clone 9E10, Roche Molecular Biochemicals) mouse monoclonal antibodies.

### Plate assay of stress sensitivity for growth

Wild-type and mutant strains of *S. pombe* were grown in YES liquid medium to OD_600_ = 0.6. Appropriate dilutions were spotted per duplicate on YES solid medium or in the same medium supplemented with different concentrations of MgCl_2_ and FK506 (*VIC* phenotype) [24], or Caspofungin. Plates were incubated at 28°C for 3–5 days, and then photographed.

### Reproducibility of results

All experiments were repeated at least three times. Relative Units (RU) for Pmk1 activation are estimated in each experiment by determining the signal ratio (as a measurement of band intensity) of the anti-phospho-P44/42 blot (activated Pmk1) with respect to the anti-HA blot (total Pmk1) at each time point. Depending on the experiment, mean relative units ± SD and/or representative results are shown. *P*-values were analyzed by unpaired Student's *t* test.

## Results

### Rho1 GTPase is a positive regulator of the CIP

**Figure 1:**
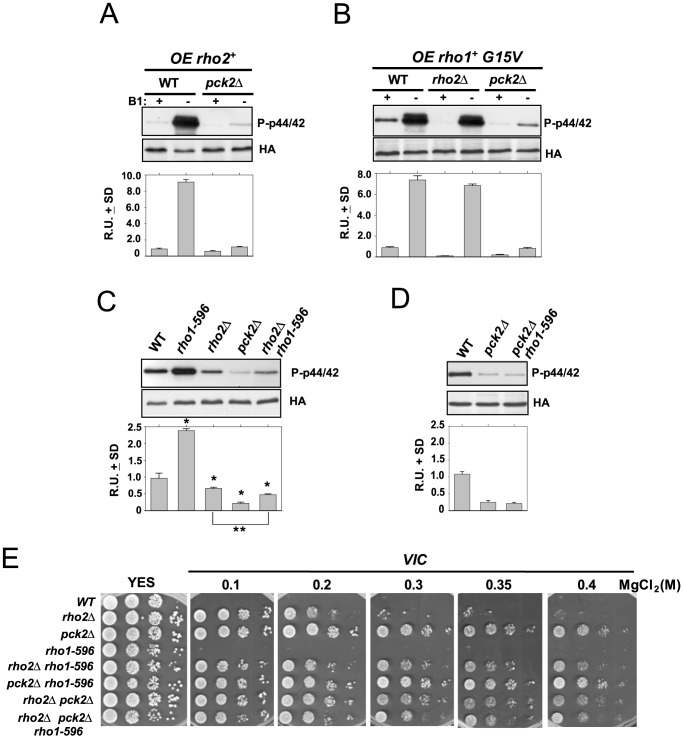
Rho1 GTPase is a positive regulator of the cell integrity MAPK pathway. (A) Strains MI200 (WT) and GB3 (*pck2Δ*) were separately transformed with pREP3X-*rho2^+^* plasmid and grown for 18 h in the presence (+B1) or absence (−B1) of thiamine. Activated Pmk1 was detected by immunoblotting with anti-phospho-p42/44 and total Pmk1 with anti-HA antibodies. (B) Strains MI200 (Control), MI700 (*rho2Δ*), and GB3 (*pck2Δ*) were separately transformed with pREP3X-*rho1+*(*G15V*) plasmid, grown for 18 h with or without thiamine, and both total and activated Pmk1 were detected as above. (C) Strains MI200 (WT), GB3 (*pck2Δ*), and LS203 (*rho1-596 pck2Δ*) were grown in YES medium to mid log-phase. Aliquots were harvested and Pmk1-HA6H was purified by affinity chromatography. Pmk1-HA6H was purified and the activated and total Pmk1 was detected as already indicated. *,*P*<0.05 in mutant strains as compared to the wild type. **,*P*<0.04 in *rho1-596 rho2Δ* cells as compared to the *rho2Δ* mutant. (D) Strains MI200 (WT), LS201 (*rho1-596*), MI700 (*rho2Δ*), GB3 (*pck2*) and LS202 (*rho1-596 rho2Δ*) were grown in YES medium to mid log-phase. And both activated and total Pmk1 was detected as above. (E) *VIC* assays for strains MI200 (WT), MI700 (*rho2Δ*), GB3 (*pck2Δ*), LS201 (*rho1-596*), GB29 (*rho2Δ pck2Δ*), LS202 (*rho1-596 rho2Δ*), LS203 (*rho1-596 pck2Δ*), and LS207 (*rho1-596 rho2Δ pck2Δ*). After growth in YES medium, 10^4^, 10^3^ or 10^2^ cells were spotted onto YES plates supplemented with 0.5 µg/ml FK506 plus 0.1, 0.2, 0.3, 0.35 or 0.4 M MgCl_2_, and incubated for 4 days at 28°C before being photographed.

Previous evidence has shown that Rho2 is a main positive regulator operating upstream of the cell integrity pathway [13], [14]. Rho2-dependent regulation of Pmk1 activity is mediated through Pck2, since the low basal Pmk1 phosphorylation level in *pck2Δ* cells is identical to that observed in the *rho2Δ pck2Δ* double mutant [14]. Also, Pmk1 hyperactivation triggered by *rho2^+^* overexpression is fully attenuated in mutants lacking Pck2 (Figure 1A). However, Pmk1 can be activated in the absence of either Rho2 or Pck2, suggesting the existence of additional regulatory elements [14]. Rho1 GTPase might modulate the activity Pmk1 by acting upstream of Pck2 because it has been described that overexpression of wild type or a constitutively active allele of *rho1^+^* (*G15V* mutant) induced a marked hyperactivation of Pmk1 (Figure 1B) [21]. While this increase was not affected by deletion of Rho2, Pmk1 activation was strongly compromised in *pck2Δ* cells (Figure 1B). Therefore, these results suggest that Rho1 is an activator of the CIP acting upstream to Pck2 in a Rho2-independent fashion. However, the existence of a low but reproducible MAPK activation in *pck2Δ* cells (Figure 1B), indicates that other elements might be targets for Rho1 during the control of Pmk1 activity (see below).

Recently we have obtained a fission yeast mutant expressing a genomic hypoactive version of Rho1 (*rho1-596* allele) [22]. *rho1-596* cells display an evident increase in basal Pmk1 activity as compared to control cells (Figure 1C) [22], suggesting that, contrary to the above findings, Rho1 might be a negative regulator of the Pmk1 pathway. We also found that Pmk1 hyperactivation in *rho1-596* cells is suppressed in a *rho2Δ* background (Figure 1C), supporting that low GTPase activity in Rho1- cells induces a cellular stress transduced to the MAPK cascade through Rho2 [22]. However, a careful examination of these experiments revealed that Pmk1 basal phosphorylation in *rho1-596 rho2Δ* cells was actually lower than in *rho2Δ* cells, and this difference was statistically significant (*P*<0.04; Figure 1C). On the contrary, basal Pmk1 activity was nearly identical in *pck2Δ* and *rho1-596 pck2Δ* cells (Figure 1D), strongly suggesting that enhanced Pmk1 activation in *rho1-596* cells is transmitted to the MAPK cascade mainly through Pck2. Deletion of members of the cell integrity pathway enables cells to grow in the presence of MgCl_2_ plus the specific inhibitor of calcineurin FK506, a feature known as the *VIC* phenotype [28]. Moreover, increasing the concentration of MgCl_2_ in the medium allows to distinguish between *rho2Δ* mutants, which display moderate Pmk1 activity, and those with very low (*pck2Δ*) or null (*mkh1Δ*, *pek1Δ*, *pmk1Δ*) MAPK activity [14]. As seen in Figure 1E, *rho2Δ* cells showed a partial *VIC* phenotype in medium supplemented with 0.2 M MgCl_2_ and became *VIC* negative in the presence of 0.3 M MgCl_2_. As expected, both wild type and *rho1-596* cells were *VIC* negative under any condition (Figure 1D). Importantly, the *VIC* phenotype in *rho1-596 rho2Δ* double mutant was markedly enhanced as compared to that shown by *rho2Δ* cells (Figure 1E), which is in good agreement with basal Pmk1 phosphorylation data (Figure 1C). As a whole, these results sustain that Rho1 GTPase is a true positive regulator of the cell integrity pathway which operates during vegetative growth in an alternative fashion to Rho2 and using Pck2 as a main target.

### Role of Rho1 during Pmk1 activation and cell survival in response to cell wall stress

**Figure 2:**
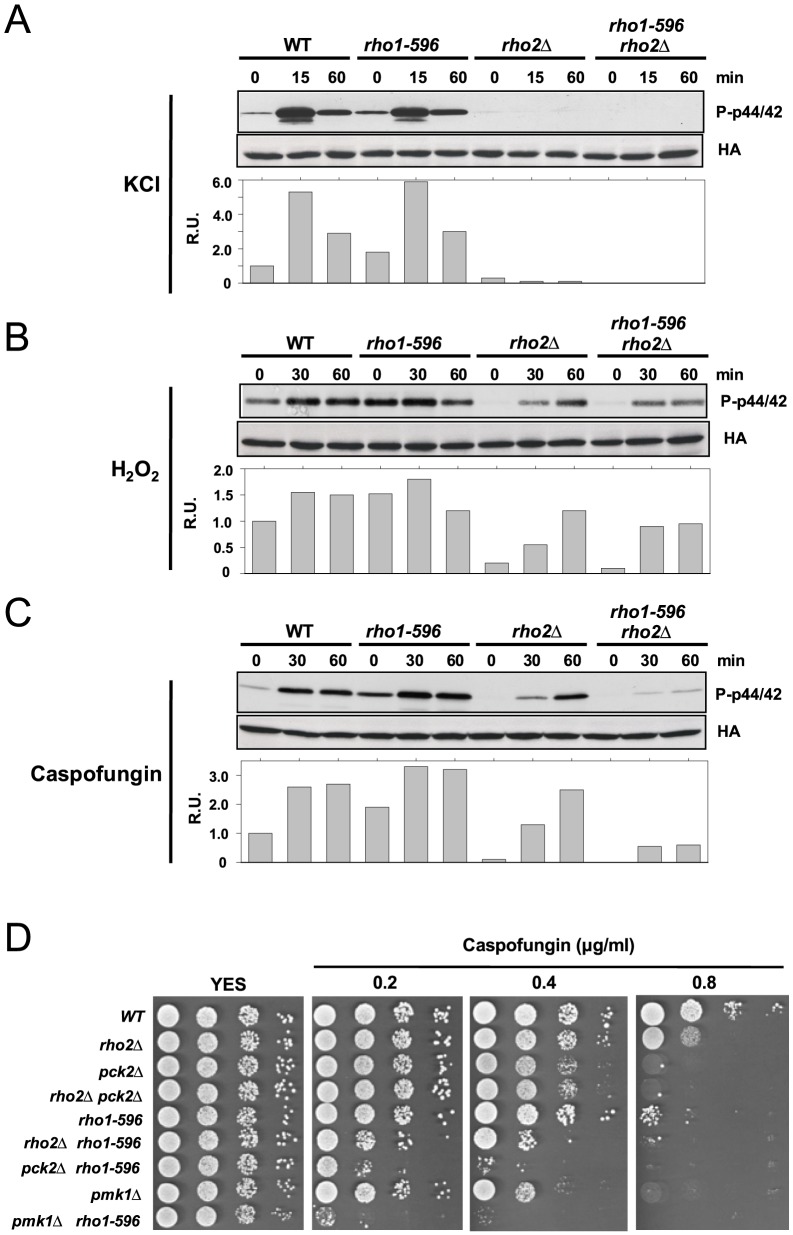
Rho1 is involved in Pmk1 activation in response to cell wall stress. Strains MI200 (*pmk1-HA6H*, WT), LS201 (*rho1-596*), MI700 (*rho2Δ*), and LS202 (*rho1-596 rho2Δ*) were grown in YES medium to mid log-phase, and treated with (A) 0.6 M KCl, (B) 1 mM H_2_O_2_, or (C) 1 µg/ml Caspofungin. At different times Pmk1-HA6H was purified and both activated and total Pmk1 were detected by immunoblotting with anti-phospho-p42/44 and anti-HA antibodies, respectively. (D) Rho1 and Pmk1 play additive roles in cell survival during cell wall stress. Strains MI200 (WT), MI700 (*rho2Δ*), GB3 (*pck2Δ*), GB29 (*rho2Δ pck2Δ*), LS201 (*rho1-596*), LS202 (*rho1-596 rho2Δ*), LS203 (*rho1-596 pck2Δ*), MI102 (*pmk1Δ*) and LS209 (*rho1-596 pmk1Δ*) were grown in YES medium, and 10^4^, 10^3^, 10^2^ and 10 cells were spotted onto YES plates supplemented with increased concentrations of Caspofungin. The plates were incubated for 4 days at 28°C before being photographed.

Pmk1 activation induced by hypo- and hyper-osmotic stress totally depends upon the signaling mediated by Rho2 (Figure 2A) [14]. On the contrary, MAPK activation triggered by oxidative (hydrogen peroxide) and cell wall (Caspofungin) stresses is only partially dependent on this GTPase (Figure 2B and C) [14]. The recognition of Rho1 as a member of the cell integrity pathway prompted us to test its role during signal transduction in response to stresses transmitted to Pmk1 in a Rho2-independent fashion. As shown in Figure 2B, Pmk1 activation in *rho2Δ* cells subjected to oxidative stress was not affected by simultaneous expression of the *rho1-596* hypoactive allele. However, in comparison to either control or *rho2Δ* and *rho1-596* single mutant cells, MAPK activation was severely compromised in cells from the *rho2Δ rho1-596* double mutant treated with Caspofungin (Figure 2C). Thus, the above results are congruent with the idea that Rho1 exerts an important role during signal transduction to the CIP in response to cell wall stress in addition to Rho2. Fission yeast mutants lacking members of the CIP pathway show a moderate (*rho2Δ*) to strong (*pck2Δ*, *pmk1Δ*) sensitivity for growth in the presence of Caspofungin [13]. In addition, the viability of *rho1-596* cells is also compromised in the presence of this cell wall stressor (Figure 2D) [22]. Interestingly, simultaneous expression of *rho1-596* aggravated the Caspofungin-sensitive phenotype in *rho2Δ*, *pck2Δ* or *pmk1Δ* cells (Figure 2D). Altogether, the above results strongly suggest that the role of Rho1 to promote cell survival during cell wall stress involves both Pmk1-dependent and -independent pathways.

### Pck1 is a positive regulator of the CIP

**Figure 3:**
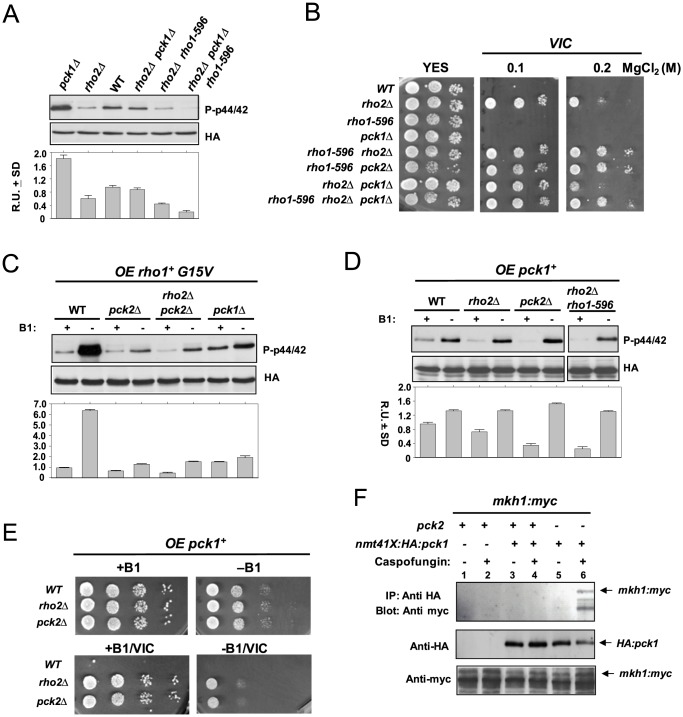
Pck1 is a positive regulator of the cell integrity MAPK pathway. (A) Strains MI200 (WT), MI700 (*rho2Δ*), GB35 (*pck1Δ*), LS206 (*rho2Δ pck1Δ pmk1-HA6H*), LS202 (*rho1-596 rho2Δ pmk1-HA6H*), and LS208 (*rho1-596 rho2Δ pck1Δ pmk1-HA6H*) were grown in YES medium to mid log-phase. Pmk1-HA6H was purified and both activated and total Pmk1 were detected by immunoblotting with anti-phospho-p42/44 and anti-HA antibodies, respectively. (B) *VIC* assays for strains MI200 (WT), MI700 (*rho2Δ*), LS201 (*rho1-596*), GB35 (*pck1Δ*), LS202 (*rho1-596 rho2Δ*), LS203 (*rho1-596 pck2Δ*), LS206 (*rho2Δ pck1Δ*), and LS208 (*rho1-596 rho2Δ pck1Δ*). After growth in YES medium, 10^4^, 10^3^ or 10^2^ cells were spotted onto YES plates supplemented with 0.5 µg/ml FK506 plus 0.1 or 0.2 M MgCl_2_, and incubated for 3 days at 28°C before being photographed. (C) Strains MI200 (WT), GB3 (*pck2Δ*), GB29 (*rho2Δ pck2Δ*), and GB35 (*pck1Δ*) were separately transformed with pREP3X-*rho1^+^*(G15V) plasmid and grown for 18 h in the presence (+B1) or absence (−B1) of thiamine. Pmk1-HA6H was purified and the activated and total Pmk1 was detected as indicated above. (D) Strains MI200 (Control), MI700 (*rho2Δ*), GB3 (*pck2Δ*), and LS202 (*Rho1-596 rho2Δ*) were separately transformed with pREP3X-*pck1^+^* plasmid, grown for 24 h with or without thiamine, and both total and activated Pmk1 were detected as above. (E) Strains MI200 (WT), MI700 (*rho2Δ*), and GB3 (*pck2Δ*) transformed with pREP3X-*pck1^+^* plasmid were grown in minimal medium, and 10^4^, 10^3^, 10^2^ or 10^1^ cells were spotted onto EMM2 medium with or without 5 µg/ml thiamine in the presence/absence of 0.5 µg/ml FK506 plus 0.2 M MgCl_2_ (*VIC*). The plates were incubated for 3 days at 28°C before being photographed. (F) Strains LS210 (*mkh1-13myc*; lanes 1 and 2, negative controls), LS211 (*nmt41:HA-pck1 mkh1-13myc*; lanes 3 and 4), and LS212 (*nmt41:Ha-pck1 mkh1-13myc pck2Δ*; lanes 5 and 6) were grown in EMM2 medium in the absence of thiamine for 24 h, and left untreated (uneven lanes), or supplemented with 1 µg/ml Caspofungin for 1 hour (even lanes). Cell extracts were immunoprecipitated (IP) with anti-Ha antibody (12CA5) and the immunocomplexes adsorbed with protein A-Sepharose. The complexes obtained were resolved by SDS-PAGE, and hybridized separately with anti-Ha and anti-myc antibodies.

We originally postulated that, contrary to Pck2, Pck1 might be a negative regulator of the CIP, since Pmk1 phosphorylation and activity result increased in *pck1Δ* cells [14]. However, the finding that the enhanced Pmk1 phosphorylation in *rho1-596* mutant cells is the result of a stress signal transmitted to the MAPK cascade via Rho2 suggested that a similar mechanism might operate for Pck1. Figure 3A indicates that deletion of *rho2+* gene alleviated the increased Pmk1 basal phosphorylation present in *pck1Δ* cells. However, the suppression was not complete, suggesting that the cell stress arising from *pck1+* deletion may be transduced to Pmk1 through additional element/s. An obvious candidate to perform such a role is Rho1, since both Pck2 and Pck1 are *in vivo* targets for this GTPase [15]. Indeed, basal Pmk1 phosphorylation in growing cells from a *rho1-596 rho2Δ pck1Δ* triple mutant further decreased as compared to *rho2Δ pck1Δ* cells (Figure 3A), and was accompanied with a enhanced *VIC* phenotype (note in Figure 3B the robust growth of *rho1-596 rho2Δ pck1Δ* cells in medium supplemented with 0.2 M MgCl_2_ as compared to *rho2Δ pck1Δ* cells). Also, we observed that Pmk1 hyperactivation induced by overexpression of the Rho1-*G15V* allele was attenuated in *pck1Δ* cells, although not as strongly as in *pck2Δ* cells (Figure 3C). Moreover, overexpression of the wild type *pck1+* allele under the control of a medium strength thiamine-repressible promoter (41X; 24 h) induced a modest but reproducible increase in Pmk1 phosphorylation in wild type cells which remained unaffected by the absence of Rho2, Pck2, or in *rho1-596 rho2Δ* cells (Figure 3D). Pck1 overexpression suppressed only partially the *VIC* phenotype of *rho2Δ* and *pck2Δ* cells (Figure 3E), suggesting that its putative role during the regulation of ionic homeostasis is not critical. Taken as a whole, these results support that Pck1 might act as a Rho1 target during signal transmission to the CIP, although its role within this pathway seems restricted to specific situations.

In spite of a previous report describing that, in contrast to Pck2, Pck1 does not interact with Mkh1 (MAPKKK) [13], the above findings allow to predict that, if Pck1 is a direct activator of the CIP, it should then associate with Mkh1 *in vivo*. We explored this possibility by performing co-inmunoprecipitation assays with exponentially growing and Caspofungin-treated strains co-expressing a genomic version of Mkh1 tagged at its C-terminus with the 13myc epitope, and a N-terminal HA-tagged version of Pck1 expressed under the regulation of the medium strength thiamine repressible promoter, both in the presence and absence of Pck2. Notably, as shown Figure 3F, we detected the Mkh1-myc fusion after Pck1 immunoprecipìtation only in Caspofungin-treated *pck2Δ* cells, but not in growing cells or strains expressing Pck2. These data suggest that the *in vivo* association between Mkh1 and Pck1 is favored during cell wall stress, and that Pck2 is then the main PKC-type kinase involved in signal transmission to the cell integrity MAPK pathway.

### Role of Pck1 during Pmk1 activation and cell survival in response to cell wall stress

**Figure 4:**
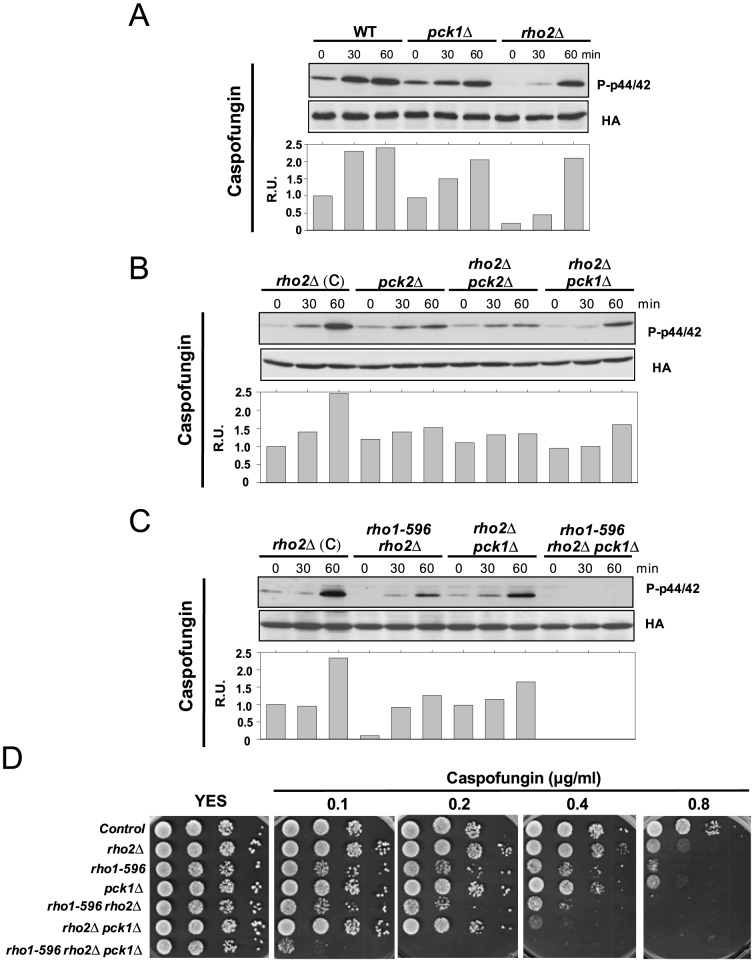
Pck1 participates in Pmk1 activation in response to specific cell wall stresses. (A) Strains MI200 (WT), GB35 (*pck1Δ*), and MI700 (*rho2Δ*); (B) MI700 (*rho2Δ*), GB3 (*pck2Δ*), GB29 (*rho2Δ pck2Δ*), and LS206 (*rho2Δ pck1Δ*); and (C) MI700 (*rho2Δ*), LS202 (*rho1-596 rho2Δ*), LS206 (*rho2Δ pck1Δ*), and LS208 (*rho1-596 rho2Δ pck1Δ*), were grown in YES medium to mid log-phase and treated with 1 µg/ml Caspofungin. At different times Pmk1-HA6H was purified and both activated and total Pmk1 were detected by immunoblotting with anti-phospho-p42/44 and anti-HA antibodies, respectively. (D) Strains described above were grown in YES medium, and 10^4^, 10^3^, 10^2^ and 10 cells were spotted onto YES plates supplemented with increased concentrations of Caspofungin. The plates were incubated for 4 days at 28°C before being photographed.

Since Pck1 appears to interact with the CIP during cell wall stress, we studied its role during signal transduction in response to this specific stimulus. Pmk1 activation in *pck1Δ* cells treated with Caspofungin was slightly lower than in control cells, whereas Rho2 absence compromised signaling particularly at early times (Figure 4A). This activation delay was similar to that observed during glucose limitation [29].The key role of Pck2 during Caspofungin-mediated response was evidenced by the minimum Pmk1 activation observed in either *pck2Δ or rho2Δ pck2Δ* cells (Figure 4B). However, the lower MAPK activation in *rho2Δ* cells was also reduced by simultaneous deletion of *pck1+*, and showed similar levels than in *rho1-596 rho2Δ* cells (Figure 4B and C). Notably, Pmk1 activation in response to Caspofungin was completely abolished in cells from a *rho1-596 rho2Δ pck1Δ* triple mutant, thus confirming that Pck1 is a member of the CIP involved in signaling during cell wall damage (Figure 4C). The Pck1-less mutant showed a moderate sensitivity to growth in the presence of Caspofungin, which was similar to that shown by the *rho1-596* mutant (Figure 4D). However, the marked growth defect of *rho1-596 rho2Δ* cells in the presence of the drug was aggravated in the *rho1-596 rho2Δ pck1Δ* triple mutant (Figure 4C). Hence, in fission yeast both Rho1 and Pck1 may act in the same pathway during the cellular defense against cell wall stress, although the possibility that Pck1 exerts its function in a Rho1-independent fashion cannot be discarded.

## Discussion

In this work we present evidence showing that in fission yeast both Rho1 GTPase and the PKC-ortholog Pck1 are members of the cell integrity MAPK pathway which promote Pmk1 activation during cell growth and cell wall stress. This signaling route functions in addition to Rho2 and Pck2, the only previously known regulators of this pathway [8], [13], [14]. Although earlier findings suggested that Rho1 might perform such a role [21], recent results pointed out into the opposite direction, since a strain expressing the hypomorphic Rho1 allele *rho1-596* displayed increased basal Pmk1 phosphorylation in growing cells [22]. However, enhanced MAPK activity likely results from a stress provoked by low Rho1 GTPase activity. Because Rho1 is an essential GTPase involved in cell wall formation and biosynthesis in fission yeast [18], the appearance of cell wall defects and the concomitant stress signaling to the Pmk1 MAPK cascade should be expected when its function results compromised. Moreover, this signal is channeled through Rho2, since both the thermosensitive and Pmk1 hyperactivated phenotypes of *rho1-596* cells are fully suppressed in the *rho1-596 rho2Δ* double mutant [22]. Importantly, in absence of Rho2, the true role of Rho1 as an alternative positive regulator of this MAPK cascade becomes evident by the increased *VIC* phenotype and the decreased basal Pmk1 phosphorylation observed in *rho1-596 rho2Δ* cells as compared to the *rho2Δ* mutant.

The fact that Pmk1 activity is still detected in Rho2-less cells in response to oxidative or cell wall stress [14] led us to hypothesize that Rho1 might positively regulate the CIP in addition to Rho2 under these conditions. We found that, contrary to *rho1-596* or *rho2Δ* cells, Pmk1 activation induced by the cell wall stressor Caspofungin became strongly limited in *rho1-596 rho2Δ* double mutant. Therefore, in fission yeast both Rho1 and Rho2 GTPases are general upstream activators of the CIP in response to cell wall damage. However, Rho1 plays a role in cell wall construction and maintenance which is different from its function as a member of the Pmk1 cascade. This is proved by the increased growth sensitivity of *rho1-596 pmk1Δ* cells to Caspofungin as compared with corresponding single mutant counterparts. Such observation is consistent with the previous demonstration that Rho1 can directly regulate the biosynthesis of (1,3) β-D-glucan and also indirectly through Pck2 and Pck1 [15]. On the other hand, our findings additionally suggest that Rho1/Rho2 independent pathways are responsible for Pmk1 activation in response to hydrogen peroxide stress. This situation is similar to recent reports showing that Pmk1 activation in the absence of glucose is transduced to the CIP in a Rho1/Rho2 independent fashion [29], and highlights the complex functional architecture for the upstream elements participating in this signaling pathway. Nevertheless, since the *rho1-596* allele is not a complete lack-of-function mutant, the possibility that residual Rho1 activity in *rho1-596 rho2Δ* cells might preferentially limit Pmk1 activation in response to some stimuli (oxidative stress, glucose absence) and not to others (cell wall stress) can not be entirely ruled out. Recently, Mtl2 and Wsc1, two plasma membrane-associated cell wall sensors, have been shown to act by turning on Rho1 activity [30]. However, Pmk1 signalling remained functional in *mlt2Δ* and *wsc1Δ* mutants subjected to osmotic and cell wall stresses, suggesting that they act independently of the CIP [30]. Thus, the upstream regulatory network of the cell integrity MAPK pathway in fission yeast is clearly more intricate than in budding yeast, where RHO1 is the only GTPase involved in signal transduction to PKC1 kinase, the sole PKC-type protein present in this organism [16], [31].

The role of Pck1 in the maintenance of cell wall integrity has not yet been clearly defined. It is well known that Pck1 and Pck2 share overlapping roles in the maintenance of cell viability and partially complement each other, with mutants lacking both kinases showing synthetic lethality [15]. Pck2 is the target for Rho1 and Rho2 during the biosynthesis of the two the main cell wall polymers in fission yeast, (1,3) β-D-glucan and α-glucan [15], and also the premier upstream activator of the Pmk1 MAPK module [14]. On the other hand, Pck1 is also targeted by Rho1 to regulate the biosynthesis of (1,3) β-D-glucan, with no apparent role in α-glucan synthesis [15], and it was considered to be either unrelated or a negative regulator of Pmk1 activity [13], [14]. Contrariwise, several evidences obtained in this work strongly suggest that Pck1 actually regulates Pmk1 activity in a positive fashion. First, Pmk1 hyperactivation elicited by overexpression of a Rho1 hyperactive allele results partially suppressed in a *pck1Δ* background. Second, ectopic expression of *pck1+* induces a moderate increase in Pmk1 phosphorylation; and third, deletion of *pck1+* in *rho1-596 rho2Δ* cells decreases basal Pmk1 phosphorylation and eliminates the remaining Pmk1 activation in response to cell wall stress. Altogether, these findings support that Pck1 participates in the regulation of Pmk1 activity during growth and in response to cell wall stress.

In agreement with previous work [13] we were unable to detect in exponentially growing cultures an *in vivo* interaction between Pck1 and Mkh1, which is the sole MAPKKK component of the CIP. Intriguingly, we confirmed the existence of a Pck1-Mkh1 association in *pck2Δ* cells treated with Caspofungin, but not in control cells expressing wild type levels of Pck2. The simplest explanation for these observations is that, whereas Pck2 is the main activator of Mkh1 under vegetative growth and in most environmental contexts, the Pck1-Mhk1 *in vivo* association is strongly limited during growth and restricted to very few situations (i.e. cell wall damage). This prediction is in agreement with the modest increase in Pmk1 phosphorylation and lack of lethality observed upon *pck1+* overexpression as compared to that induced by either *pck2+* or *rho2+* [21]. However, although limited, the role of Pck1 as a positive reinforce of the cell integrity pathway is biologically relevant since Pmk1 activity becomes partially abrogated in *pck1Δ* cells under the conditions described above. Hence, the unstable nature of Pck1-Mkh1 association might be the underlying reason for the impossibility to detect *in vivo* interaction under conditions where Pck1 actually regulates the MAPK cascade and in the presence of Pck2.

**Figure 5:**
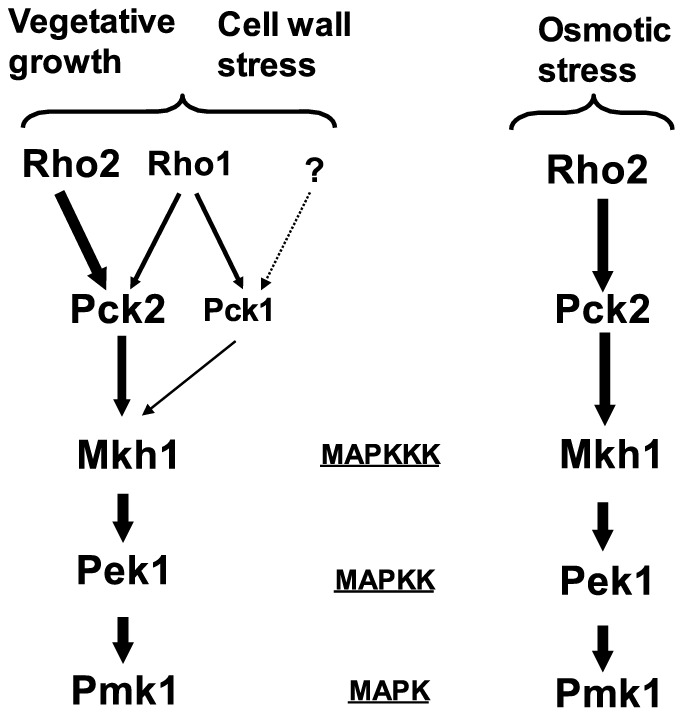
In fission yeast, Rho1 GTPase and PKC ortholog Pck1 are upstream activators of the cell integrity MAPK pathway in addition to Rho2 and Pck2 under specific situations. Sizes of protein names and line arrows intend to show the relative significance of each component of the cascade during signaling to the MAPK module in unstressed growing cells and in cell wall and osmotic stressed cells (please see text for details).

Another important question raised in this work refers to the epistatic relationship between Rho1 and Pck1 during the regulation of the CIP. The fact that Pck1 is an *in vivo* target of Rho1 and that Pmk1 hyperactivation induced by Rho1 overexpression is partially suppressed in *pck1Δ* cells, suggests that Pck1 activity might be solely mediated by this GTPase. On the contrary, the complete blockage of Pmk1 activity/activation in *rho1-596 rho2Δ pck1Δ* triple mutant cells as compared to what happens in *rho1-596 rho2Δ* cells points to the existence of additional elements regulating Pck1 activity in a Rho1-independent fashion. This is in agreement with our observation that the growth sensitive phenotype of *rho1-596 rho2Δ* cells in the presence of Caspofungin was enhanced by simultaneous deletion of *pck1+*. Although none of the above possibilities can be completely excluded, it appears likely that the low GTPase activity in *rho1-596 rho2Δ* cells might be sufficient to promote Pck1-dependent Pmk1 activation to a certain degree. In this context, the role of Pck1 as a Rho1 target during signaling to the CIP becomes evident in the absence of the kinase. In summary, based on this work and previous studies, we propose that Rho1 and Pck1 are *bona fide* functional members of the cell integrity MAPK pathway in fission yeast in addition to Rho2 and Pck2 (Figure 5). Rho1 and Rho2 support Pmk1 basal activity during vegetative growth, primarily via Pck2, whereas the contribution of Pck1 is then small. Under cell wall perturbations Rho1 activates the MAPK cascade through either Pck2 or Pck1, in addition to the major activating stimulus provided via Rho2-Pck2. This model emphasizes the branched nature of the upstream signal network that regulates the CIP and attempts to establish functional relationships among the various members involved in such intricate signaling pathway.
